# The Use of Glycomacropeptide in Dietary Management of Phenylketonuria

**DOI:** 10.1155/2016/2453027

**Published:** 2016-05-30

**Authors:** Osama K. Zaki, Lamia El-Wakeel, Yasmin Ebeid, Hanan S. Ez Elarab, Aisha Moustafa, Nayera Abdulazim, Hala Karara, Ahmed Elghawaby

**Affiliations:** ^1^Genetics Unit, Pediatrics Hospital, Ain Shams University, Ramsis Street, Abbasia, Cairo 11566, Egypt; ^2^College of Pharmacy, Ain Shams University, Abbasia, Cairo, Egypt

## Abstract

Dietary therapy is the most common therapy applied in treatment of Phenylketonuria (PKU) with restriction of intake of most natural proteins that are rich in Phenylalanine (Phe). Recently, it has been claimed that caseinoglycomacropeptide (GMP), derived of whey, may be used to replace the amino acid formulae (AAF).* The Aim of Work*. To study the feasibility of use of GMP for partial replacement of artificial formula in treatment of children with PKU.* Methods*. Ten patients with PKU were included in the study. They received the recommended daily allowances of protein in the form of AAF or a combination of AAF and GMP. The percent of intake of GMP in phases 1 and 2 was 50% and zero%, respectively.* Results*. The median and interquartiles of phenyl alanine Phe levels phase were not significantly different in phases I and II, 376 (167–551) *μ*mol/L versus 490 (289–597) *μ*mol/L, respectively. Phenylalanine/tyrosine ratio, amino acids, and other laboratory data showed no significant difference between the two phases.* Conclusion*. GMP may be used to replace 50% of the protein intake to improve the nutritive value and palatability of diet and to provide a more satisfactory diet. No toxicity or side effects were reported in patients on that regimen.

## 1. Introduction

Classic Phenylketonuria (PKU) is a metabolic disorder caused by a deficiency in the production of the hepatic enzyme Phenylalanine Hydroxylase (PAH). Dietary therapy with restriction of dietary Phe intake remains the mainstay of therapy for PAH deficiency, requiring a decrease in the intake of natural protein and replacing it with a protein (amino acid mixture) source devoid of Phe. Dietary manipulation required to maintain appropriate blood Phe levels needs frequent modification to respond to growth, life stages, concurrent illness, and comorbidities. An experienced metabolic physician and nutritionist team should manage this therapy. It is important to monitor blood Phe and tyrosine (Tyr) levels and to ensure that other nutritional requirements are also being met [1, 2]. The amount of natural foods allowed in a diet are adjusted to ensure that the level of Phenylalanine in the blood is kept within a safe range 240 to 360 *μ*mol under 12 years of age and 240–600 *μ*mol for PKU patients over 12 years old [3].

The main source of protein in the diet of patients with PKU is supplied as artificial amino acid formulae (AAF) that are usually prepared of mixture of amino acids that lack Phenylalanine. One of the main obstacles to compliance of PKU patients to treatment is that the amino acid mixtures used in the formulae usually have a bitter taste and strong odor [4, 5].

Recently, it was found that a protein derived of whey called caseinoglycomacropeptide (GMP), a side product of cheese manufacture, has a low content of Phe. This makes it a source for a near-natural food recipes for PKU patients [6, 7]. It has the advantage of being of lower osmolarity than the regular amino acids formula. In addition mass production from cheap whey would make it a suitable alternative for developing countries [8].

Ney and coworkers (2008) found that PKU mice showed adequate growth and level of Phe in plasma and brain after intake of a modified diet with GMP as the major source of protein. Their data supported the use of GMP supplemented with indispensable amino acids as an alternative source of dietary protein for individuals with PKU [7]. GMP also provided a physiological source of low Phe dietary protein that promotes growth and attenuated the metabolic stress and bone fragility compared to a low Phe amino acid supplemented diet in PKU mice [9, 10].

A short duration study was done on 11 PKU patients who adhered to the amino acid formula diet for four days and then switched to the GMP diet for the following four. No adverse health problems were found, and 10 of 11 subjects claimed to prefer the GMP diet, making the bottom line of this study that short-term use of GMP is safe and acceptable [11]. The same workers described the experience of an adult patient with PKU who volunteered to consume an all-GMP diet for 10 weeks [12]. The authors mentioned that not only did the subject enjoy the palatable GMP-fortified diet that supplied most of his daily protein, but the amount of Phenylalanine in his blood actually went down after intake of these items for a couple of weeks.

*Aim of the Work*. Most of the above-mentioned studies were done on mice models or adults; the follow-up period was only few days. So far, there have been no long-term studies on human subjects with PKU who received GMP supplemented diet. This study was done to investigate the feasibility of partial replacement of amino acid formulae with GMP for nutritional therapy in children with PKU.

## 2. Patients and Method

This study is a prospective, self-controlled, small-scale clinical trial involving classical PKU patients attending Ain Shams Genetics Unit who accepted to be enrolled in the study program and informed about plan of study and concerns. The work was carried out in accordance with the Code of Ethics of the World Medical Association (Declaration of Helsinki) for experiments involving human after approval of the Ethical Committee of Ain Shams University and the guardians of patients should sign the informed consent after elaborate explanation of the nature and procedures of the study.

*Inclusion Criteria*. Patients with PKU were recruited from those attending at the Metabolic Clinic, Genetics Unit of Ain Shams University (GUASH). They had to be more than two years old and compliant to treatment for at least two months prior to start of study.

*Exclusion Criteria*

- Patient with atypical PKU.
- PKU patient suffering from convulsions, osteoporosis, and other metabolic, liver, or kidney diseases.
- Noncompliant PKU patient and patient who choose not to be involved or to continue the study to its end.
- Patients who may prove to be allergic to GMP or who develop abnormal liver, kidney function, other unpredicted responses, or untoward reaction after the intake of GMP.

### 2.1. Methods

Study period was divided into two phases according to the percentage of protein in cheese made of GMP (the GMP was purchased from Davisco Foods International, Inc., 11000 West 78th Street, Suite 210, Eden Prairie, MN 55344. GMP Cheese was made of GMP and butter in a ratio of 1 : 1 with added salt. The mixture is kept in the refrigerator till used in the form of spread cheese. The amount of recommended GMP was divided into doses that are added to the three main meals of the patient (breakfast, lunch, and dinner)) and amino acid formulae in diet as shown in Table 1. Throughout the study, all patients received the same total protein intake to match the recommended protein requirement for their age and weight [13]. They were allowed a free intake of low protein foods (low protein breads and pasta, cookies, etc.) as well as controlled amounts of natural foods with low Phenylalanine content. The diet regimen was closely followed up by experienced physicians aiming at keeping Phenylalanine level between 240 and 360 *μ*mol for children under 12 years of age and 240–600 *μ*mol for PKU patients over 12 years old [3].

*All patients were subjected to the following:*

- Weekly follow-up of the level of amino acid/acylcarnitine profiles including Phe and Phe/Tyr ratio as well as 11 other amino acids was carried out. This was done using equipment tandem mass spectrometry (Acquity LC-MS/MS from Waters Inc.) and nonderivatised amino acid/acyl-carnitine kits from Chromsystems Inc.
- Monthly follow-up laboratory investigation for liver function tests (ALP, AST, ALT, albumin, bilirubin, and GGT), BUN, creatinine in blood and complete blood count (CBC) was carried out.
- A questionnaire was filled up by the patient or guardian at the end of each phase to evaluate the satisfaction of patients regarding satiety and palatability of the GMP diet compared to regular diet with AAF.

### 2.2. Statistical Analysis

The median and mean levels of Phenylalanine Phe/Tyr ratio as well as other 13 amino acids that were tested during the study phases were analyzed using repeated measures ANOVA test. Baseline data measurements were used to compare the changes in outcome laboratory measures.

The laboratory data of the first two weeks of each phase were excluded of the statistical analysis to avoid the possibility of overlap of response to the diet regimens.

## 3. Results

Ten patients were included in this study (six boys and four girls). Their age ranged from four to sixteen years. The median age and interquartile were 6.73 and (5.02–11.79).

The median level of Phe at the start of the study was 521.5 (232.25–833).

**Figure 1:**
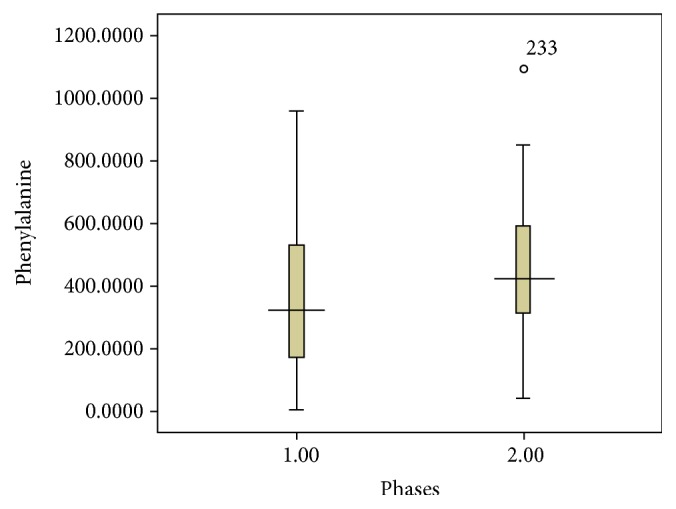
The level of Phe in the phases of the study in ten patients (70 samples in phase I and 66 samples in phase II).

It was possible to achieve adequate control of Phe in phase I and the level of Phe was comparable to that achieved with 100% synthetic formulae as a source for low Phe protein. The median of Phe level during phase I was 376 (167–551) *μ*mol/L which was not significantly different from the 490 (289–597) *μ*mol/L that was encountered in phase II (Figure 1).

**Figure 2:**
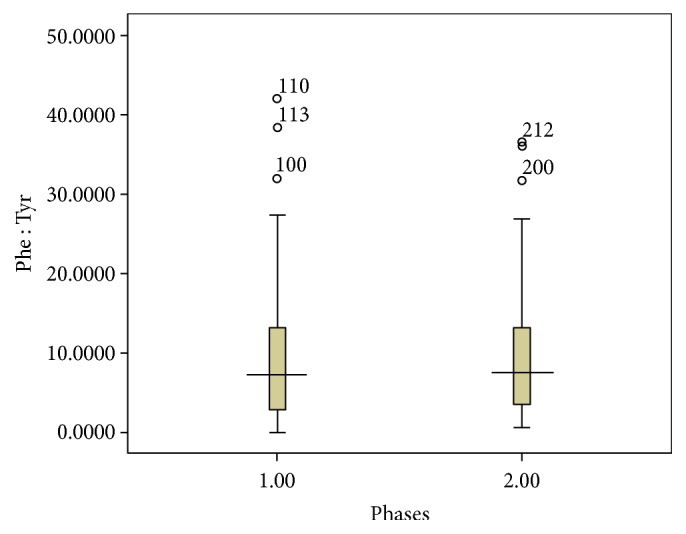
Level of Phe/Tyr ratio in the phases of the study in ten patients (70 samples in phase I and 66 samples in phase II).

In addition, the Phe/Tyr ratio was not significantly different between the two phases (Figure 2).

**Figure 3:**
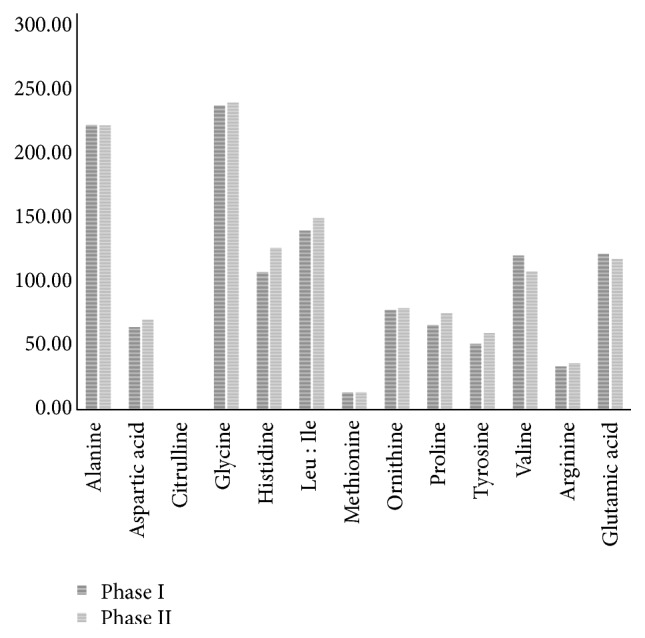
Comparison of measured AA levels in the two phases (70 samples in phase I and 66 samples of in phase II).

The levels of other amino acids were generally lower in phase I than other phases with significantly low level of aspartic acid and citrulline (Figure 3). On the other hand, the levels of all amino acids in phases I and II were not significantly different.

Other laboratory investigations showed no significant difference between parameters in phases of the study.

Throughout the study, all patients preferred the diet regimen that is supplemented with GMP over the classical AAF due to better taste and satiety.

## 4. Discussion

GMP has unique characteristics that may qualify to replace amino acid formulae. It has been recommended for management of PKU as a replacement of amino acid supplement.

So far, few studies have shown that GMP may be suitable for the management of PKU [11, 12]. A recent randomized controlled clinical trial including 30 adults compared the effects of GMP and AA medical foods in the nutritional management of PKU. Results showed that subjects rated GMP medical foods as more acceptable and convenient than AA formula. There were no adverse events associated with either the AA or GMP diets. Subjects reported gastrointestinal symptoms and persistent hunger with the AA diet that improved with the GMP diet [14].

Daly and coworkers have reported a higher level of Phe and Phe/tyrosine ratio in a group of 9 children who received 66% of their protein intake in the form of GMP and 33% of L-AA. They concluded that GMP could only partly replace L-AA supplements [15].

The current study tested the use of GMP as a partial source of low Phe protein in children with PKU.

During Phase I, the combination of equal amounts of GMP and AAF proved to be successful in achieving a level of control of the disease comparable to that achieved with the classic AAF regimen. Close follow-up of several laboratory parameters did not show any drawbacks of this regimen (Table 2). In fact, the level of albumin was higher in this phase than in phase II. This may be the result of better digestibility and metabolism comparable to that was reported by Solverson and coworkers in mice with PKU [9].

In addition, the 50% GMP regimen was preferred by all patients as they were able to eat the more palatable “cheese” made of GMP. They also expressed a better feeling of satiety. This is commensurate with earlier observation of MacLeod and coworkers who reported a better satiety and lower level of appetite stimulating hormone (ghrelin) after intake of GMP in breakfast [16].

## 5. Conclusion

The above findings show that incorporation of up to 50% of the protein as GMP into food products is safe and decreases the stress caused by the limited food stuff that are made solely of AAF. GMP is an excellent addition to diet of children with PKU when supplemented with AAF. On the contrary unsupplemented GMP is not suitable as the sole source of low Phe protein.

The use of a side product of whey such as GMP in the management of PKU should increase compliance, improve outcome, and reduce cost of treatment especially in developing countries where the cost of commercial medical foods composed of AA may be unaffordable. On the contrary, whey is readily available as a cheap side product of cheese. However, this approach requires the development of large-scale facilities for production of GMP to provide the cheaper alternative or adjuvant to the synthetic amino acid based foods.

## Figures and Tables

**Table 1:** The source of low Phenylalanine (low Phe) diet during the phases of the study.

	Duration	Source of low Phe protein supplement	
		GMP	AAF
Phase I	9 weeks	50%	50%
Phase II	9 weeks	0%	100%

**Table 2:** Levels of follow-up laboratory parameters in all phases.

		Number of samples	Mean	Std. deviation	Min.	Max.	*F*	Sig.
	
S_urea	Baseline	10	20.6	7.66	6	30	.353	0.7
	Phase I	8	17.63	5.18	8	23		
	Phase II	6	19.67	9.75	10	37		
	Total	24	19.37	7.32	6	37		

S_creat	Baseline	10	.44	.15	.2	.7	.44	0.6
	Phase I	9	.38	.13	.2	.6		
	Phase II	3	.37	.12	.3	.5		
	Total	22	.41	.14	.2	.7		

S_Albumin	Baseline	9	4.6	.35	4.2	5.2	.224	0.8
	Phase I	8	4.5	.52	4	5.3		
	Phase II	5	4.52	.31	4	4.8		
	Total	22	4.5	.40	4	5.3		

S_ALT	Baseline	10	15.6	6.18	9	25		
	Phase I	9	16.6	4.38	11	26	.09	0.9
	Phase II	5	15.8	5.44	11	25		
	Total	24	16	5.2	9	26		

S_AST	Baseline	10	23	9.8	7.	37	.49	0.6
	Phase I	9	26	5.6	15	32		
	Phase II	5	26.4	4.6	20	31		
	Total	24	24.8	7.4	7	37		

S_Hb	Baseline	8	11.9	1.51	10.2	14.1	.63	0.5
	Phase I	10	11.49	1.5	10.1	14.7		
	Phase II	5	12.38	1.32	10.7	14.1		
	Total	23	11.84	1.45	10.1	14.7		
